# Lysine-specific histone demethylase 1A (LSD1) in cervical cancer

**DOI:** 10.1007/s00432-020-03338-z

**Published:** 2020-07-28

**Authors:** Daniel Beilner, Christina Kuhn, Bernd P. Kost, Julia Jückstock, Doris Mayr, Elisa Schmoeckel, Christian Dannecker, Sven Mahner, Udo Jeschke, Helene Hildegard Heidegger

**Affiliations:** 1 Department of Obstetrics and Gynaecology, LMU Munich, Marchioninistraße 15, 81377 Munich, Germany; 2 Department of Pathology, LMU Munich, Thalkirchner Str. 56, 80337 Munich, Germany; 3 Department of Obstetrics and Gynaecology, University Hospital Augsburg, Stenglinstr. 2, 86156 Augsburg, Germany

**Keywords:** LSD1, Epigenetic modification, DNA methylation, GPER, Cervical cancer, Survival

## Abstract

**Purpose:**

Demethylation of DNA through enzymes like LSD1 showed a crucial impact on different kind of cancers. Epigenetic modifications in cervical cancer are still not fully investigated nevertheless of high interest for a therapeutic use.

**Methods:**

Tumor samples of 250 cervical cancer patients were immunochemically stained and evaluated based on Immunoreactive Score. Results were statistically analyzed for clinical and pathological parameters.

**Results:**

Our patient collective showed a disadvantage for 10-year survival for patients with a strong expression of LSD1 in the cytoplasm of cervical cancer cells. The results of the correlational analysis further revealed a negative correlation of LSD1 to G-protein coupled estrogen receptor (GPER).

**Conclusions:**

Epigenetic changes through enzymes like LSD1 may also be of interest for patients with cervical cancer. A combined therapy with other proteins relayed to cervical cancer like GPER might be of interest for future investigations.

## Background

Cervical cancer is with about 570,000 new cases in the year 2018, the fourth most frequent cancer after breast, colorectum and lung carcinoma in women worldwide. It represents about 6.6% of all female cancers. About 311,000 deaths occurred in 2018 caused of cervical cancer (World Health Organization [WHO (2020a)].About 90% of the cervical cancer deaths occur in low and middle- income countries. Prevention, early diagnosis, effective screening and treatment programmes are essential to reduce the mortality rate of cervical cancer (World Health Organization [WHO (2020b)]. The two main histologic subtypes are squamous cell carcinoma with about 80% and adenocarcinoma with about 20% (Aviel-Ronen et al. 2016).

Cervical cancer is usually caused by persistent high-risk Human Papillomavirus (HR-HPV) infection, with human papillomavirus (HPV) being the most common sexually transmitted biological agent. About 85% of anogenital cancers in men and women are related to HPV infection (Leite et al. 2020).

HPV belongs to the Papillomaviridae family and is a circular, non-enveloped double-stranded DNA virus. Many subtypes have already been identified (Leite et al. 2020; Goodman 2015). There is a sub-classification of HPV infections of the genital tract in low risk, found mainly in genital warts-, and high risk HPV. HPV-6, HPV-11, HPV-40, HPV-42, HPV-43, HPV-44 and HPV-53 belongs to the low risk subtypes. The high risk types like HPV-16, HPV-18, HPV-31, HPV-33, HPV-35, HPV-39, HPV-45 and others are frequently associated with invasive cervical cancer (Munoz et al. 2003).

In the last years, the epigenetic processes in cancer causation, progression and treatment played an important role. Epigenetic abnormalities like the DNA methylation are leading candidates for the diagnosis, prognosis and the development of markers for cancer detection (Baylin and Jones 2011). Epigenetic therapy like histone modification was already successful in treating hematopoietic malignancies (Liu et al. 2017).

LSD1 (Lysine Specific Demethylase 1) works as a histone demethylase and as a transcriptional corepressor by demethylating histone H3 lysine 4, which is associated to active gene transcription (Shi et al. 2004).

LSD1 plays a role in cellular processes like cell proliferation (Lan et al. 2008) and stem cell pluripotent regulation (Whyte et al. 2012) and a dysregulation was associated with human cancer development (Amente et al. 2013; Gu et al. 2020).

Overexpression of LSD1 was already found in several types of solid tumors like prostate cancer, bladder cancer, neuroblastoma, lung cancer, sarcomas, colorectal or hepato-carcinomas (Amente et al. 2013; Hayami et al. 2011). Elevated LSD1 expression correlates with tumor progression and negative clinical outcomes (Liu et al. 2017). An inhibition or knock down of LSD1 was found to suppress cell growth, invasion and migration in solid tumors like non-small cell lung cancer (Lv et al. 2012). For some cancer types, LSD1 has also been proposed as a biomarker (Amente et al. 2013).

Regarding the high levels of LSD1 which is associated with tumor progression, in the last years this protein was an interesting target for drug discovery; it was proposed that epigenetic drugs targeting LSD1 could be used for the therapy of cancer (Amente et al. 2013).

To investigate the role of LSD1 in cervical cancer more studies are needed, so the aim of this study is to identify epigenetic modifications in cervical cancer to improve future knowledge for diagnostics and therapy of cervical cancer.

## Methods

### Patients

Our study analysed 250 paraffin embedded cervical cancer samples from patients of the Department of Obstetrics and Gynaecology of the Ludwig-Maximilians University of Munich (LMU) between 1993 and 2002. Our samples included only the two most frequent histological subtypes, squamous cell carcinoma and adenocarcinoma. Other subtypes were not chosen for their relatively small number of cases. Table 1 presents an overview of clinical and pathological parameters from our specimens.

**Table 1:** Clinical and pathological parameters for age, pN-status, pT-status, FIGO-classification, grading, histological subtype and recurrence

	No./total no	%
Age		
≤ 50 years	141/250	56.4
> 50 years	105/250	42.0
N/A	4/250	1.6
pN		
Negative	151/250	60.4
Positive	97/250	38.8
N/A	2/250	0.8
pT		
T1	111/250	44.4
T2	128/250	51.2
T3/4	9/250	3.6
FIGO		
I	64/250	25.6
II	48/250	19.2
III	37/250	14.8
IV	7/250	2.8
N/A	94/250	37.6
Grading		
G1	20/250	8.0
G2	143/250	57.2
G3	78/250	32.2
N/A	9/250	3.6
Histological subtype		
Squamous carcinoma	202/250	80.8
Adenocarcinoma	48/250	19.2
Recurrence (within 235 months)		
None	190/250	76.0
≥ 1	58/250	23.2
N/A	2/250	0.8

### Immunohistochemistry

For analysing the expression of LSD1 in our study group we performed an immunohistochemical staining. In the first step in this process paraffin embedded and formalin fixed tumor samples of 3 µm were prepared on microscope slides. Our samples were first deparaffined with Roticlear and then washed in 100% ethanol. With the next step, we blocked endogenous peroxidase with 3% methanol/H2O2 followed by a treatment in descending alcohol levels for rehydration. The samples were washed in distilled water and then cooked in a pressure cooker covered in a sodium-citrate buffer (pH = 6.0) for 5 min by a maximum heat of 100 °C. Afterwards the samples were cleaned again in distilled water before washing the slides in PBS-buffer. For a valid staining, we first treated our tumor samples with a blocking solution to keep unspecific hydrophobic binding as low as possible and then incubated our slides with the primary LSD1 antibody (Anti-LSD1). After an incubation time of 16 h by 4 °C, our slides were washed in PBS-buffer, treated with post-block solution and then covered in HRP-polymer. After washing the slides again in PBS-buffer the tumor samples were stained with DAB and directly counterstained with Haemalaun. To complete the staining process our samples were dehydrogenated again in a rising alcohol series.

To determine whether a patient had a low or high expression of LSD1 the Immunoreactive Score (IRS) was used and each tumor sample was rated from 0 (no expression) to 12 (very high expression). Like established, IRS was calculated from intensity (0 = not stained; 1 = low intensity; 2 = moderate intensity, 3 = high intensity) multiplied by the percentage rate of stained cells (0 = not stained; 1 = 1–10%; 2 = 11–50%; 3 = 51–80%; 4 ≥ 81%).

### Statistics

To analyse our collected data, we created a database using IBM Statistics version 25 (Amrok, NY, USA). For all significant results, *p* was required to be < 0.05. Median expression for various clinical and pathological parameters was studied. Cumulative survival time, cox regression and correlations on this patient group were calculated.

## Results

### LSD1 staining in cervical cancer

**Fig. 1:**
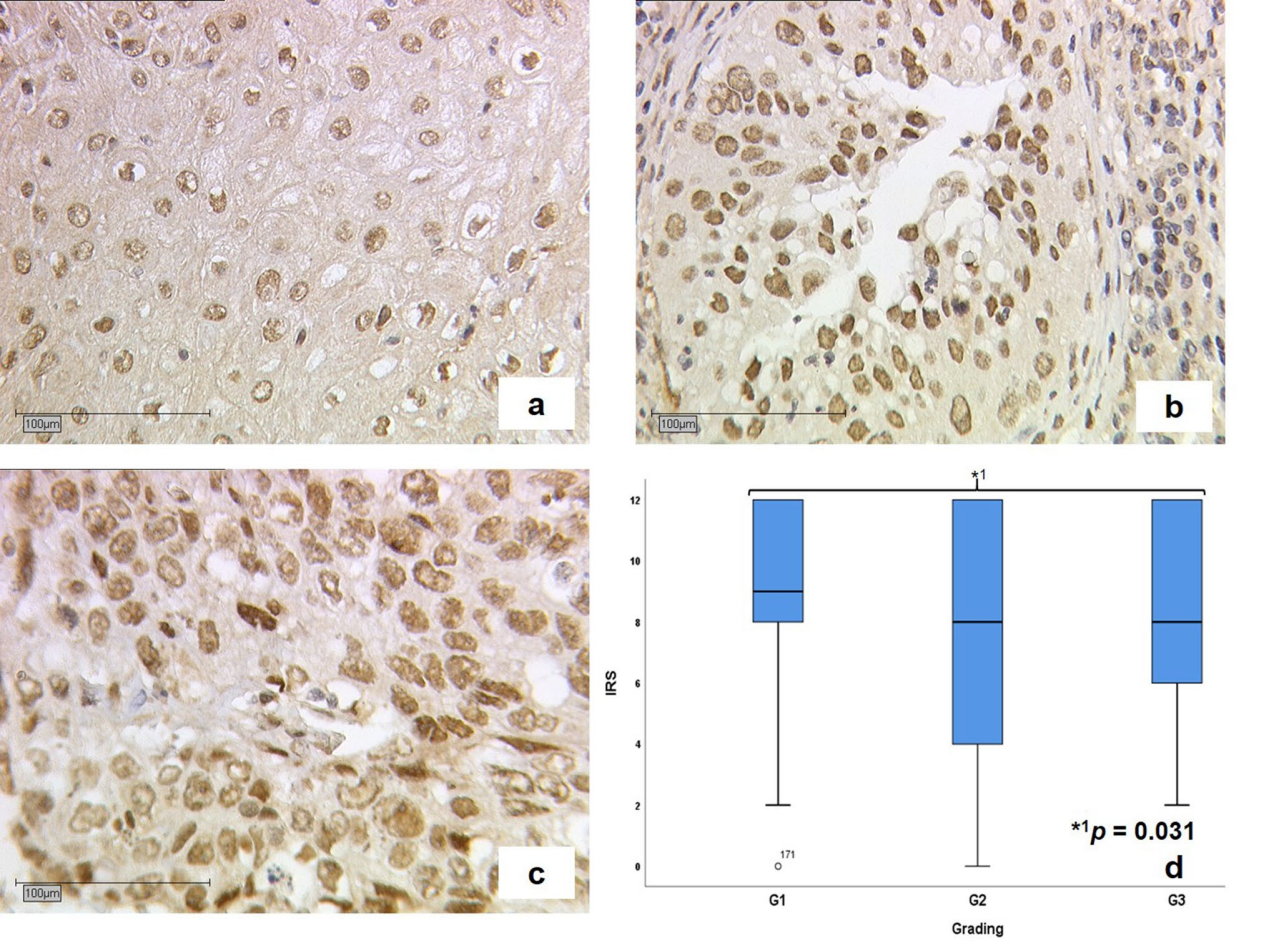
Illustration of immunohistochemical staining results for grading. Stained cervical cancer samples with a median cytoplasmic IRS for grading G1 of 9 (**a**), G2 of 8 (**b**) and G3 of 8 (**c**). **d** presents boxplots with median IRS of 9 for grading G1 and a median IRS of 8 for grading G2 and G3 in cytoplasm. The asterisk (*) indicates significant lower expression of LSD1 in patients with higher Grading

The median cytoplasmic IRS of the staining was 8 compared with a median IRS of 12 in the nucleus. 1.8% of the cases showed no expression in the cytoplasm and 0.5% no detectable staining in the nucleus. 28.0% showed a low expression in the cytoplasm while 72.0% presented a high expression in the cytoplasm. In comparison, 6.6% presented a low and 93.4% a high expression in the nucleus. The median cytoplasmic IRS for squamous carcinoma and for adenocarcinoma was 8 and the nuclear median IRS was 12 for both histological subtypes. Depending on tumour grading appeared a median cytoplasmic IRS of 9 for grading G1 and a median IRS of 8 for grading G2 and G3 tumours (Fig. 1) while median nuclear IRS was 12 for each grading. According to tumour size each t-status showed a median IRS of 8 in cytoplasm and 12 nuclear for T1 and T2. Tumour size T3/4 had a median IRS of 8, both for nucleus and cytoplasm. Patients with (N +) or without (N-) lymph node metastasis had a median IRS of 8 in the cytoplasm and a nuclear IRS of 12. Cytoplasmatic median IRS for FIGO I-III tumours was 8 while FIGO IV tumours showed a median IRS of 4 in cytoplasm. All cases from FIGO I to FIGO IV presented a median IRS of 12 in the cell nucleus. Table 2 illustrates the median IRS for the expression of LSD1 for histological subtype, grading, T-status, N-status and FIGO classification separately in cytoplasm and nucleus.

**Table 2:** Immunohistochemical results for LSD1 staining. Illustration of median IRS for expression of LSD1 for histological subtype, grading, T-status, N-status and FIGO classification separately in cytoplasm and nucleus

	Cytoplasm	Nucleus
Median IRS	8	12
Expression		
No expression	1.8%	0.5%
IRS 1–5	28.0%	6.6%
IRS 6–12	72.0%	93.4%
Histological subtype (IRS)		
Squamous carcinoma	8	12
Adenocarcinoma	8	12
Grading		
G1	9	12
G2	8	12
G3	8	12
T-status		
T1	8	12
T2	8	12
T3	8	8
N-status		
N( +)	8	12
N(−)	8	12
FIGO		
I	8	12
II	8	12
III	8	12
IV	4	12

### Correlation analyses of LSD1 staining with other parameters in cervical cancer

For further investigation of LSD1 as a prognostic factor in cervical cancer, we analysed our data bank for correlations of LSD1 expression and other parameters. There was a significantly negative correlation between LSD1 expression and GPER (*p* = 0.009).

### Correlation of LSD1 expression in cervical cancer and survival

**Fig. 2:**
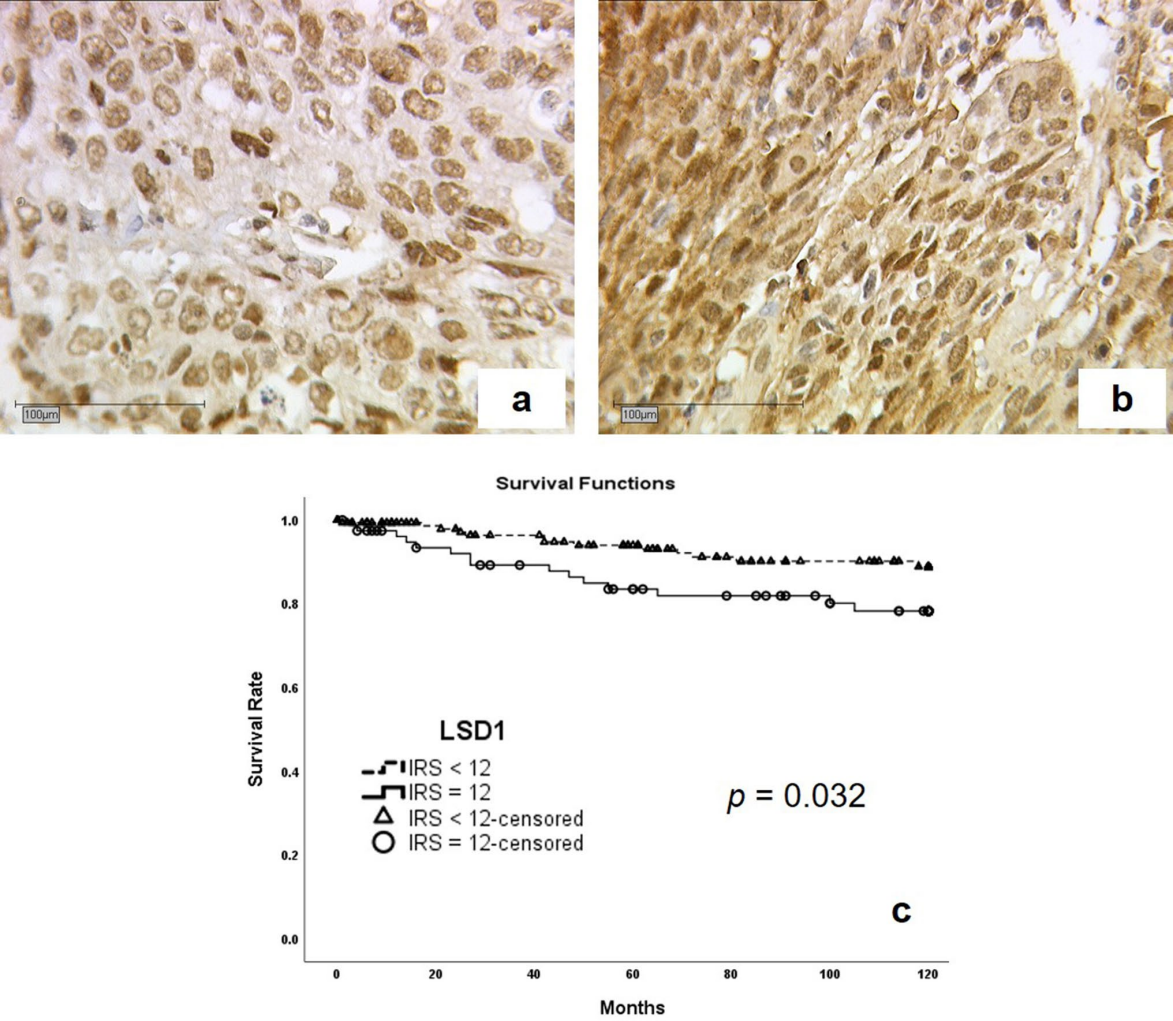
Illustration of survival analysis. Cervical cancer tissue stained with anti-LSD1 with a low cytoplasmic IRS of 8 (**a**) compared to a sample with high cytoplasmic IRS of 12 (**b**). Complemented by Kaplan–Meier analyses for 10-year-survival (**c**): high cytoplasmatic LSD1 expression (IRS = 12; **○-**solid line) compared to low expression (IRS < 12; ▲-dashed line) regarding relapse-free survival (*p* 0.032)

With a median age of 47 years, we recorded patients in a range from 20 to 83 years and a median survival time of 100 months. A very strong IRS (IRS = 12) of the cytoplasm for LSD1 showed a disadvantage for 10-year overall survival (*p* = 0.032). Within 120 months, if an IRS of 12 was seen 10% more women died compared to women with a lower IRS. A high LSD1 IRS-score was related with a 9.4 months shorter median survival time (Fig. 2).

### Cox regression

In order to evaluate independent histological parameters for survival in our specimens we used a multivariate cox-regression analysis. As presented in Table 3, histological subtype (*p* = 0.001), pN-status (*p* = 0.009) and expression of LSD1 (*p* = 0.037) were independent prognostic factors for overall survival.

**Table 3:** Cox-regression. Multivariate cox-regression presenting histological subtype (p = 0.001), pN-status (p = 0.009) and expression of LSD1 (p = 0.037) as independent prognosticators for overall survival

	Significance	Hazard Ratio of Exp (B)	Lower 95% CI of Exp (B)	Upper 95% CI of Exp (B)
Histology	0.001	3.212	1.579	6.532
pN	0.009	2.593	1.275	5.275
FIGO	0.689	1.015	0.945	1.089
Grading	0.095	1.611	0.920	2.821
LSD1 cytoplasm (IRS = 12)	0.037	2.071	1.046	4.099

## Discussion

This study was designed with the aim of assessing expression of LSD1, an enzyme responsible for DNA demethylation and subsequent epigenetic modifications to develop further knowledge in the challenging task of clinical diagnostics and therapy of cervical cancer. The current study found that a high expression of LSD1 in cervical cancer tissue was a significant disadvantage in 10-year-survival in our patient collective. It is also interesting to note that we found a significant negative correlation of LSD1 expression to G-Protein Coupled Estrogen Receptor (GPER).

Many epigenetic mechanisms have been discovered to modify gene expression leading this machinery to play a fundamental role in biological diversity of cells (Taby and Issa 2010). In the same way, epigenetic aberrations are a central part of cancer development including DNA methylation (Baylin and Jones 2011). LSD1 is a histone lysine demethylase and functions as an important transcriptional corepressor by demethylating H3K4me2/me1 and H3K9me2/me1 (Shi et al. 2004). Prior studies have already described that dysregulation of LSD1 can influence cancer development: Overexpression of LSD1 correlates with poor prognosis in various cancer types like bladder cancer (Hayami et al. 2011), colon cancer (Jie et al. 2013) or hepatocellular carcinoma (Kim et al. 2019). Our result are contrary to a previous study by Liu et al. (2017) who could not find a statistically significant difference in overall nor in tumor free survival for cervical cancer patients with a high or low expression of LSD1 (Liu et al. 2017). However, our findings instead broadly support the work of previous studies about LSD1 linking an overexpression of cytoplasmic LSD1 to a disadvantage in 10-year survival also for patients with cervical cancer. Despite that the previous mentioned study found that ectopic expression of LSD1 in cervical cancer cells increases invasion and metastasis (Liu et al. 2017) and confirms the picture of LSD1 as a negative factor in cervical cancer. Besides its specific demethylase activity, LSD1 has been increasingly described to play a role in a wide range of cellular processes (Gu et al. 2020) like cell differentiation (Lan et al. 2008) or migration and invasion of cancer (Ambrosio et al. 2017). It is therefore likely that LSD1 will become an interesting target for anticancer treatment (Yang et al. 2018).

Another important finding was a negative correlation of LSD1 expression to GPER expression, a seven-transmembrane-domain receptor that mediates non-genomic estrogen related signaling (Xu et al. 2019). During the last years, estrogen was continuously discovered to be deeply involved in cancer progression in different kinds of cancers (Chan et al. 2017; Hsu et al. 2017; Yu et al. 2017). Estrogen signals are suggested to play a crucial role in development of cervical cancer (Zhang et al. 2015). Thus, non-genomic estrogen effects performed by GPER has become an interesting subject to study (Xu et al. 2019). Our study group described in 2014 GPER as a positive prognostic factor in ovarian cancer with reduced overall survival for patients with a strong GPER intensity (Heublein et al. 2014). In accordance with the previous results another study from our group confirmed GPER to be associated with improved recurrence-free and overall survival also in cervical cancer (Friese et al. 2018). A possible explanation for this negative correlation of LSD1 and GPER might be that tumor suppressor inactivation originates mainly in genetic and epigenetic mechanisms including methylation of the promoter to supress the gene’s transcription (Osborne et al. 2004; Payne and Kemp 2005). In accordance with this, another study has demonstrated that GPER expression was influenced through methylation and demethylation of the promoter in breast cancer patients (Weissenborn et al. 2014). It is therefore likely that a connection exists between both proteins—LSD1 and GPER. However, an explanation for this finding remains unanswered at present and can only be hypothesized. Nevertheless, a combined treatment of LSD1 inhibitors with other therapeutic targets (Fang et al. 2019), already supposed by Fang et al. (2019), may be also of interest for future investigations of LSD1 and GPER in cervical cancer.

The purpose of the current study was to describe epigenetic modifications in cervical cancer. This study has shown that very strong expression of cytoplasmic LSD1 showed a disadvantage in 10-year overall survival (*p* = 0.032) in our patient collective. The second major finding was a significant negative correlation between LSD1 expression and GPER (*p* = 0.009). Overall, this study strengthens the idea that epigenetic modifications are also meaningful in cervical cancer and in particular LSD1 may play an important role with a negative influence on patients’ survival. Furthermore, this study has raised important questions about connections of LSD1 and GPER. Many studies have shown that GPER can mediate multiple signaling pathways (Xu et al. 2019) and also LSD1 interacts together with many proteins (Fang et al. 2019). Future studies on LSD1 and GPER are therefore recommended to increase more knowledge of these two proteins as highly interesting therapeutic targets.

## Data Availability

The dataset used during the current study is available from the corresponding author on reasonable request.
